# The Effectiveness of Gastrectomy With Chemoradiotherapy Among Stage IV Gastric Adenocarcinoma: A Population-Based Analysis

**DOI:** 10.3389/fonc.2020.00630

**Published:** 2020-04-28

**Authors:** Shuchun Li, Lu Zang

**Affiliations:** ^1^Department of Surgery, Ruijin Hospital, Shanghai Jiaotong University School of Medicine, Shanghai, China; ^2^Shanghai Minimally Invasive Surgery Center, Shanghai, China

**Keywords:** gastric cancer, metastasis, gastrectomy, chemoradiotherapy, survival

## Abstract

**Objectives:** The strategy for the treatment of stage IV gastric cancer remains controversial. The objective of this study was to assess whether tumor resection is beneficial to survival in gastric cancer patients with incurable stage IV disease.

**Methods:** This is a retrospective cohort study of gastric cancer patients in the Surveillance, Epidemiology, and End Results (SEER) database between 2010 and 2015. Due to the baseline bias, 1:1 propensity score matching (PSM) was used in this cohort. Patients were grouped by treatment, (1) gastrectomy with chemoradiotherapy (CRT), or (2) CRT only, and a Cox proportional hazards regression model was used to identify predictors of survival. Overall survival was compared between the two groups using the Kaplan-Meier method.

**Result:** After propensity score matching, 162 stage IV gastric cancer patients diagnosed from 2010 to 2015 were identified. Among these patients, half underwent gastrectomy with CRT, while the others received CRT only. The median overall survival rates were 22 months from the date of surgery for the gastrectomy with CRT group and 9.0 months for CRT only group. In the multivariable Cox regression analysis, surgery was associated with a significant improvement in overall survival [hazard ratio (HR) of death = 0.31, 95% confidence interval (CI) = 0.21–0.46, *P* < 0.0001].

**Conclusion:** In conclusion, stage IV gastric cancer is still a fatal disease. This population-based study found that compared with CRT alone, CRT with gastrectomy may be associated with a survival benefit in patients with metastatic GC. In selected patients' survival can be prolonged when the primary tumor is removed. Prospective, randomized trials are required to determine the best strategy for metastatic GC and to describe the characteristics of the selected patients.

## Introduction

Gastric cancer (GC) is an aggressive cancer and the third leading cause of cancer-related death worldwide (1). Since it is usually diagnosed when the tumor is locally advanced or metastatic, it has a poor prognosis. However, the standard treatment strategy for metastatic gastric cancer remains controversial. Many clinical trials have proven that combination chemotherapy improves the overall survival (OS) and quality of life of metastatic gastric cancer compared that in patients treated with supportive care (2, 3). For patients with a good general condition, current practice guidelines recommend palliative chemotherapy in the European Society for Medical Oncology (ESMO) guidelines (4) and chemoradiotion or systemic therapy in the National Comprehensive Cancer Network (NCCN) guidelines (5). Due to the poor prognosis, it is crucial to look for innovative methods or the appropriate combination of treatments.

The value of surgery in metastatic GC remains controversial. Recently, REGATTA, a randomized controlled trial, has denied the effectiveness of palliative gastrectomy for metastatic GC (6). However, some studies indicated that many patients with unresectable tumors survived for a long period when they underwent curative resection after chemotherapy. Curative surgery after chemotherapy is called as conversion surgery. It is defined as a surgical treatment aiming at R0 resection after systemic therapy in initially unresectable tumors (7). This approach has been shown to be a potential option for some metastatic GC patients.

The aim of this population-based cohort study was to determine the efficacy of chemoradiotherapy with gastrectomy and whether it could prolong survival in patients with stage IV gastric cancer.

## Materials and Methods

### Data Source

A retrospective cohort study was carried out using the Surveillance, Epidemiology, and End Results (SEER) database^1^, which is a population-based cancer registry covering ~34.6% of the U.S. population. The SEER database has collected cancer incidence, prevalence, and survival data from 18 registries of the U.S. since 1973 (www.seer.cancer.gov). The SEER database includes data on patient demographics, cancer site, histologic type, stage, dates of diagnosis and survival. SEER^*^Stat version 8.3.5 was used to extract the patient data. The chemotherapy and radiation therapy (RT) status was obtained after an additional authorization and informed the potential bias related to these data (8).

### Patient Selection

**Figure 1:**
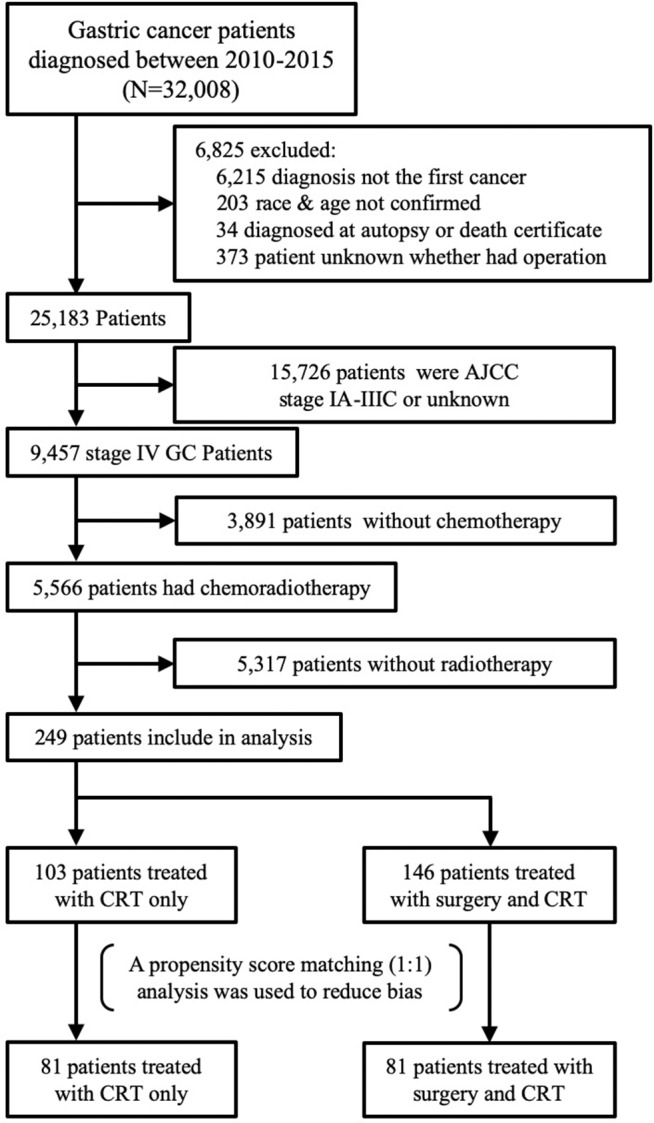
Flowchart of data extraction from SEER database.

Patients with gastric adenocarcinoma diagnosed in 2010–2015 were included in this study. Histologically diagnosed cases were identified by the specific codes of the International Classification of Diseases for Oncology, 3rd edition (ICD-O-3), including 8140/3, 8144/3, 8145/3, 8255/3, 8260/3, 8480/3, 8481/3, and 8490/3. The primary sites with ICD-O-3 topography codes from C16.0 to C16.9 were used in this study. The workflow for patient selection is shown in Figure 1. We identified 32,008 patients 18 years or older with gastric cancer. Among these, 6,215 patients were excluded because GC was not the initial diagnosis, and we subsequently also removed patients who were diagnosed at autopsy or from death certificate or who were missing baseline information. To prevent the limitation of missing treatment records, we only enrolled patients who had both chemotherapy and radiotherapy. Finally, 249 cases were enrolled for further analysis.

### Statistical Analysis

Chi-square tests were used to compare categorical variables. In an observational study, a propensity score matching analysis can be used to balance the distribution of observed baseline covariates between treated and untreated subjects and reduce the bias of selection (9, 10). By applying propensity score matching to adjust for group differences in this cohort, we first used demographic parameters, including age, sex, race, tumor location, primary site invasion depth, regional lymph nodes involved, Lauren classification and marital status, to create a logistic regression model. Then, every patient had a propensity score, which was utilized to match between the CRT with surgery group and the CRT only group (1:1 matching). The median overall survival duration was measured by the Kaplan-Meier method. The survival durations in the CRT group and the CRT with surgery group were compared by the log-rank test. Multivariable Cox proportional hazards regression models were assessed to determine the factors that were associated with survival. Statistical analyses were performed with SPSS, version 23 (IBM Corporation, Armonk, NY, USA), and R, version 3.5.1 (R Foundation for Statistical Computing, Vienna, Austria. https://www.R-project.org/). All statistical tests were two-sided, and *p* < 0.05 was considered statistically significant.

## Results

### Demographics and Clinical Parameters of the Cohort

There were 32,008 gastric adenocarcinoma patients extracted from the SEER database from 2010 to 2015. Finally, we identified 249 patients who met the inclusion criteria (Figure 1). Among the unmatched cohort, 103 underwent CRT only, and 146 had CRT with gastrectomy. Chi-square tests revealed a significant difference between the two groups. Compared with the CRT group, the CRT with gastrectomy group had a higher proportion of patients with more lymph node metastasis, especially for N3 grade (30.82 vs. 3.88%; *p* < 0.001). The primary site of the lesion was more likely located in the lower third of the stomach in the CRT with gastrectomy group (22.6 vs. 5.83; *p* < 0.001) (Table 1). The mean number of regional lymph nodes examined in the CRT with gastrectomy group was 18.40 ± 13.08. Approximately 43% of the patients had more than 15 lymph nodes examined. For the data on the surgical methods, the extent of gastrectomy was classified as total or partial/subtotal gastrectomy. The information on distant metastasis is shown in Table 2. The reasons for diagnosing stage IV gastric cancer varied, including liver involvement (23.29%), distant lymph nodes (22.49%), brain involvement (11.24%), bone involvement (13.65%), and lung involvement (12.05%). A propensity score matched analysis was used to partially reduce the baseline imbalance between the groups. Finally, 81 pairs of patients were generated by PSM one-to-one matching. For the patients who underwent surgery, 39 (48.15%), and 42 (51.85%) underwent total and partial/subtotal gastrectomy, respectively. A detailed comparison of the demographics and clinical characteristics of unmatched and matched patients is shown in Table 1.

**Table 1:** Clinical characteristics of patients who were diagnosed with stage IV gastric cancer involved in study.

	**Patient characteristics in raw data**			**Patient characteristics after propensity score weighting**		
	**CRT only *n* = 103**	**Gastrectomy + CRT *n* = 146**	***p***	**CRT only *n* = 81**	**Gastrectomy + CRT *n* = 81**	***p***
**Age at diagnosis (years)**			0.924			0.926
20–39	12 (11.65)	14 (9.58)		10 (12.35)	10 (12.35)	
40–59	43 (41.75)	64 (43.84)		34 (41.98)	30 (37.04)	
60–79	46 (44.66)	64 (43.84)		35 (43.21)	39 (48.15)	
>=80	2 (1.94)	4 (2.74)		2 (2.47)	2 (2.47)	
**Sex**			0.883			0.863
Male	73 (70.87)	101 (69.19)		58 (71.60)	57 (70.37)	
Female	30 (29.13)	45 (30.82)		23 (28.40)	24 (29.63)	
**Race**			0.224			0.240
White	77 (75.76)	102 (69.86)		62 (76.54)	61 (75.31)	
Black	7 (6.80)	20 (13.70)		3 (3.70)	8 (9.88)	
Others	19 (18.45)	24 (16.44)		16 (19.75)	12 (14.81)	
**T stage**			<0.001			<0.001
T1	15 (14.56)	6 (4.11)		15 (18.52)	2 (2.47)	
T2	3 (2.91)	12 (8.22)		3 (3.70)	5 (6.17)	
T3	28 (27.18)	60 (41.10)		27 (33.33)	38 (46.91)	
T4	17 (16.50)	63 (43.15)		14 (17.28)	31 (38.27)	
TX	40 (38.83)	5 (3.42)		22 (27.16)	5 (6.17)	
**N Stage**			<0.001			0.001
N0	29 (28.16)	21 (14.38)		16 (19.75)	18 (22.22)	
N1	49 (47.57)	40 (27.40)		40 (49.38)	28 (34.57)	
N2	11 (10.68)	38 (26.03)		11 (13.58)	20 (24.69)	
N3	4 (3.88)	45 (30.82)		4 (4.94)	14 (17.28)	
NX	10 (9.71)	2 (1.37)		10 (12.35)	1 (1.23)	
**Location**			<0.001			0.165
Cardia & Fund	74 (71.84)	58 (39.73)		54 (66.67)	44 (54.32)	
Body	10 (9.71)	11 (7.53)		9 (11.11)	8 (9.88)	
Antrum & Pylorus	6 (5.83)	33 (22.60)		6 (7.41)	15 (18.52)	
Others	13 (12.62)	44 (30.14)		12 (14.81)	14 (17.28)	
**Year of diagnosis**			0.108			0.245
2010–2011	27 (26.21)	57 (39.04)		24 (29.63)	32 (39.51)	
2012–2013	38 (36.89)	44 (30.14)		29 (35.80)	20 (24.69)	
2014–2015	38 (36.89)	45 (30.82)		28 (34.57)	29 (35.80)	
**Lauren classification**			0.154			0.170
Intestinal	78 (75.73)	94 (64.38)		64 (79.01)	58 (71.60)	
Diffuse	24 (23.30)	49 (33.56)		17 (20.99)	20 (24.69)	
Others	1 (0.97)	3 (2.05)		0 (0)	3 (3.70)	
**Extend of gastrectomy**			–			–
Total	–	67 (45.89)		–	39 (48.15)	
Partial/sub-total	–	79 (54.11)		–	42 (51.85)	
**Regional node examined**			–			–
<15	–	59 (40.41)		–	31 (38.27)	
15–25	–	45 (30.82)		–	27 (33.33)	
>25	–	27 (18.50)		–	14 (17.28)	
UK	–	15 (10.27)		–	9 (11.11)	

*In the tables, the number in parentheses is the constituent ratio*.

**Table 2:** Type of distant metastasis in the cohort before matching.

**Distant metastasis organ involved**	**Patient number^**a**^*N* = 25183**	**Patient number^**b**^*N* = 249**
Liver	3909 (15.52)	58 (23.29)
Distant lymph nodes	2960 (11.75)	56 (22.49)
Lung	1369 (5.44)	30 (12.05)
Bone	1208 (4.80)	34 (13.65)
Brain	189 (0.75)	28 (11.24)

a *The gastric cancer patients extracted from SEER database*.

b *The patients enrolled in this study. In the tables, the number in parentheses is the constituent ratio*.

### Survival Outcomes

**Figure 2:**
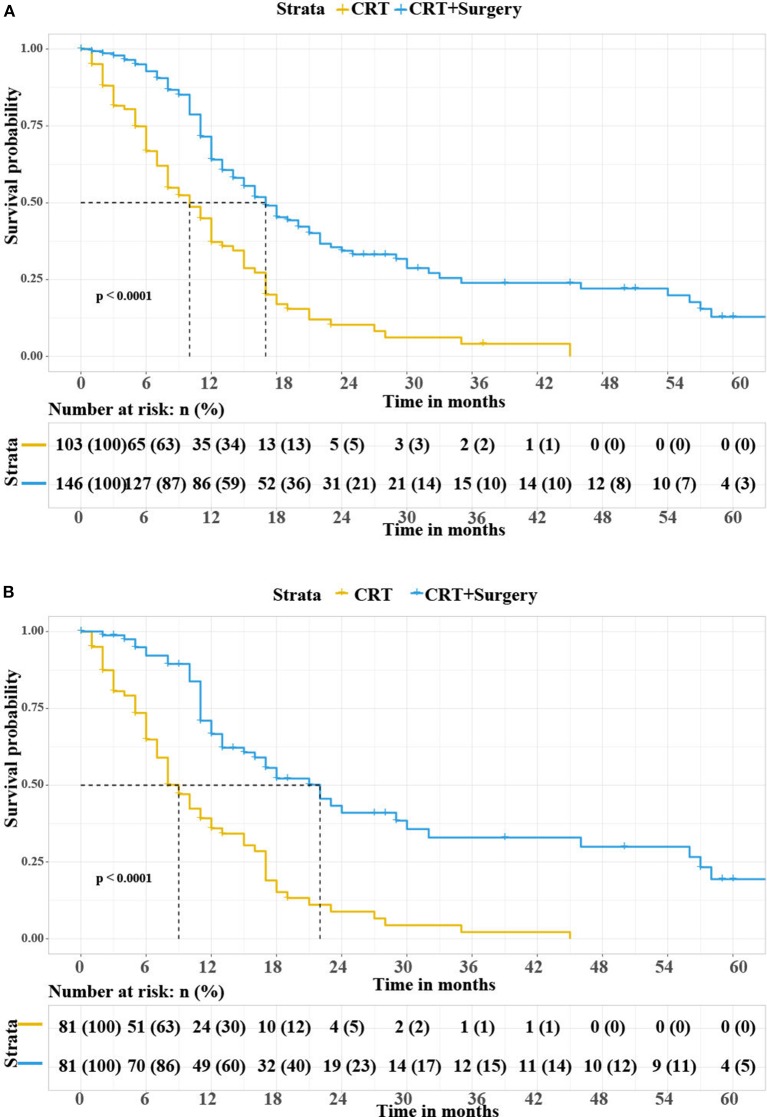
**(A)** Overall survival rate according to the treatment modalities in all patients. **(B)** Overall survival rate according to the treatment modalities in matched patients. CRT, chemoradiotherapy.

In the unmatched cohort, the median overall survival was 10 months for patients in the CRT group vs. 17 months for patients in the CRT with gastrectomy group. The Kaplan-Meier curves for overall survival are shown in Figure 2A. The log-rank test showed that the CRT with gastrectomy group had better overall survival than the CRT only group (*p* < 0.0001). After matching, the results were similar between the two groups (Figure 2B). The CRT with gastrectomy group had a 13-month longer median overall survival than the CRT only group (22 vs. 9 months for OS, *p* < 0.0001 log-rank test).

### Evaluation of Prognostic Factors

**Figure 3:**
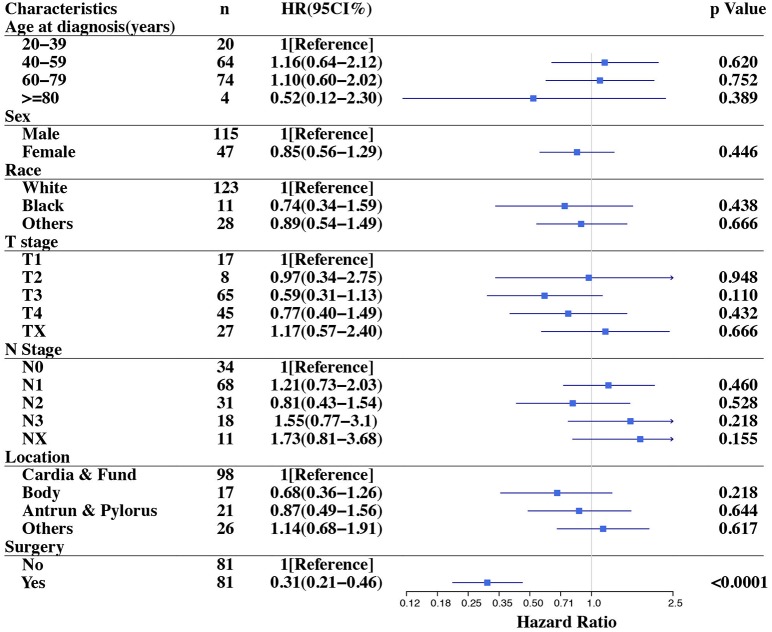
Forest plot of overall survival stratified analysis in the matched cohort.

The multivariate analysis of all patients indicated that surgery (HR = 0.23, 95% CI 0.14–0.36, *p* < 0.0001) and primary site located in antrum & pylorus (HR = 1.82, 95% CI 1.00–3.30, *p* = 0.05) were related to reduced mortality (Table 3). In the 81 paired cases, the Cox proportional hazards model was used to evaluate the prognostic factors for overall survival. Surgery was the only independent prognostic factor for OS (HR = 0.31, 95% CI 0.21–0.46, *p* < 0.001). The influence of surgery on mortality remained robust with adjustment for age, sex, race, tumor invasion depth, lymph node metastasis and primary site location. The details are illustrated in Figure 3.

**Table 3:** Multivariable cox regression analysis predicting mortality risk for metastatic GC both in unmatched and matched cohorts.

	**Unmatched cohort**			**Matched cohort**		
**Characteristics**	***n***	**HR (95CI%)**	***p-*Value**	***n***	**HR (95CI%)**	***p-*Value**
**Age at diagnosis (years)**						
20–39	26	1[Reference]	NA	20	1[Reference]	NA
40–59	107	1.28 (0.73–2.24)	0.39	64	1.16 (0.64–2.12)	0.62
60–79	109	1.27 (0.71–2.28)	0.42	74	1.10 (0.60–2.02)	0.75
>=80	7	0.92 (0.29–2.95)	0.89	4	0.52 (0.12–2.30)	0.39
**Sex**						
Male	174	1[Reference]	NA	115	1[Reference]	NA
Female	75	0.85 (0.58–1.24)	0.39	47	0.85 (0.56–1.29)	0.45
**Race**						
White	179	1[Reference]	NA	123	1[Reference]	NA
Black	27	1.05 (0.59–1.86)	0.86	11	0.74 (0.34–1.59)	0.44
Others	43	0.85 (0.53–1.37)	0.51	28	0.89 (0.54–1.49)	0.67
**T stage**						
T1	21	1[Reference]	NA	17	1[Reference]	NA
T2	15	1.74 (0.72–4.25)	0.22	8	0.97 (0.34–2.75)	0.95
T3	88	1.07 (0.56–2.04)	0.84	65	0.59 (0.31–1.13)	0.11
T4	80	1.13 (0.56–2.33)	0.71	45	0.77 (0.40–1.49)	0.43
TX	45	0.90 (0.47–1.72)	0.76	27	1.17 (0.57–2.40)	0.67
**N Stage**						
N0	50	1[Reference]	NA	34	1[Reference]	NA
N1	89	1.22 (0.76–1.95)	0.41	68	1.21 (0.73–2.03)	0.46
N2	49	1.22 (0.69–2.17)	0.49	31	0.81 (0.43–1.54)	0.53
N3	49	1.74 (1.00–3.04)	0.05	18	1.55 (0.77–3.1)	0.22
NX	12	1.24 (0.61–2.55)	0.55	11	1.73 (0.81–3.68)	0.16
**Location**						
Cardia & Fund	132	1[Reference]	NA	98	1[Reference]	NA
Body	21	0.85 (0.44–1.65)	0.63	17	0.68 (0.36–1.26)	0.22
Antrum & Pylorus	39	1.82 (1.00–3.30)	0.05	21	0.87 (0.49–1.56)	0.64
Others	57	2.10 (1.20–3.68)	0.01	26	1.14 (0.68–1.91)	0.62
**Surgery**						
No	103	1[Reference]	NA	81	1[Reference]	NA
Yes	146	0.23 (0.14–0.36)	<0.0001	81	0.31 (0.21–0.46)	<0.0001

## Discussion

Stage IV gastric cancer remains a lethal disease, and the median overall survival of metastatic or unresectable GC is ~4.6–13.1 months, as reported in previous studies (11–13). In this population-based analysis, the medial OS was 14 months, which is similar to that in a previous report. Interestingly, our investigation provides evidence of a strong association between surgery and decreased overall mortality in metastatic gastric cancer patients who received chemoradiotherapy in a large population-based study. The sensitivity analysis showed that surgery was a prognostic factor for overall survival in both the unmatched and matched cohorts. Gastric cancer disseminates principally through hematic flow or peritoneal spread. The most common metastatic distribution is to the liver and peritoneal surfaces. In previous reports, the overall rates of metastasis to the liver and peritoneum were 9.9–18.7% and 12.3%, respectively, (14, 15). In our study, the hepatic metastatic rate was 15.52%, which is similar to that in a previous study. However, there is little information on other metastatic sites in the SEER database, especially peritoneal metastasis.

The primary aim of treatment for stage IV GC is to delay disease progression and relieve symptoms such as tumor-related hemorrhage or obstruction. Systemic therapy is the primary treatment for metastatic gastric cancer. Surgery is only performed when bleeding or obstruction occurs (16). However, whether the addition of gastrectomy to chemotherapy improves survival for metastatic GC remains controversial. The Dutch Gastric Cancer Group reported that palliative resection may increase the survival rate (8.1 vs. 5.4 months, *p* < 0.001) in patients with incurable GC, especially in patients with only one metastatic site who are under 70 years old (17). A retrospective study including 288 patients also showed that the median overall survival rates were 12 months and 7.8 months for patients with and without primary tumor resection, respectively (*p* < 0.001) (18). Leonardo et al. used the GIRGC database and analyzed stage IV unresectable tumors. These tumors became resectable after chemotherapy. Further analysis showed that these patients could benefit from radical gastrectomy. More than one type of metastatic lesion was the main prognostic factor in these patients (HR 4.41, 95% CI 1.72–11.3, *p* = 0.002) (19).

These results indicate that tumor burden reduction was correlated with prolonged OS in patients with metastatic GCs. Moreover, circulating tumor cells (CTCs) are the tumor cells left from the primary site, and they enter the bloodstream. It has recently been a topic of interest in clinical cancer research (20). In other solid tumors, such as colorectal cancer (21) and ovarian cancer (22), a reduction in tumor burden was related to longer survival. Recent research on CTCs in gastric cancer provides some evidence for the positive effects of tumor resection because the OS is significantly lower for patients in whom CTCs are identified than for those without them (23).

The REGATTA trial, an open label, randomized, phase 3 trial, was designed to determine the value of gastrectomy in unresectable advanced GC, providing the highest level of evidence about this question (6). This study demonstrated that gastrectomy followed by chemotherapy did not show any survival benefit compared with chemotherapy alone. This conclusion was adopted in the new version of the Japanese gastric cancer treatment guidelines (16). Reduction surgery is not recommended for GC with a single non-cured factor. Although this study represents the highest level of evidence for gastrectomy for metastatic GC, there remain some limitations that deserve discussion. First, this study started in 2008. The chemotherapy regimen used in this study was S-1 plus cisplatin, which is the standard treatment for advanced GC in East Asia (24). However, with the development of chemotherapy, it has been showed that SOX (S-1 plus oxaliplatin) is a preferable regimen in terms of the safety profile (13). Second, the gastrectomy arm in this study had neither D2 lymphadenectomy nor adjacent organ resection, which suggested that it did not achieve R0 resection. D2 lymphadenectomy has been the standard procedure for resectable advanced GC for a long time (25). At the same time, previous studies demonstrated that R0 resection was a significant independent predictor of overall survival in patients who underwent conversion surgery (26, 27). D2 lymphadenectomy is related to higher post-operative mortality and morbidity, which may have negative effects on stage IV GC patients. Even so, R0 resection is important for prolonging OS. Furthermore, in the subgroup analyses of overall survival, the median number of chemotherapy cycles was decreased in gastrectomy with chemotherapy group compared with the chemotherapy alone group in patients with upper-third tumor (3 vs. 6 cycles). All of the points mentioned above had side effects on achieving the positive results for the trial. Besides, in the chemotherapy alone group, 5 patients underwent curable gastrectomy and get a long-term survival since complete disappearance of incurable factors after chemotherapy. Therefore, the value of gastrectomy in patients with metastatic GC should not be denied absolutely.

Conversion surgery is defined as a surgical treatment aiming at R0 resection after systemic therapy for tumors that were initially incurable (28). In recent years, positive progress for conversion surgery has been made in clinical trials. AIO-FLOT3 is an II-phase clinical study which is designed to investigate the efficacy of chemotherapy and surgery in patients with advanced gastric cancer (29). The study consisted of 3 arms. A total of 51 patients with resectable gastric cancer were included in arm A, who underwent radical surgery after 4 cycles of FLOT neoadjuvant chemotherapy and were treated with 4 cycles of FLOT chemotherapy after surgery. A total of 60 patients with localized metastatic gastric cancer were included in arm B. The localized metastasis refers to single organ metastasis with or without retroperitoneal lymph node metastasis. The patients in arm B received at least 4 cycles of FLOT chemotherapy and proceeded to surgery if it was possible to achieve a R0 resection for the primary tumor and metastatic lesions after re-evaluation. Otherwise systemic chemotherapy will be continued (8 cycles in total). A total of 127 patients with extensive metastasis were included in arm C, who underwent at least 8 cycles of FLOT palliative chemotherapy. The study endpoint was overall survival (OS). Finally, with a median follow-up time of 28.6 months, more than half of the patients in arm A were still alive. 36 (60%) patients in arm B underwent surgery, and their overall survival was significantly longer than that of arm C (22.9 vs. 10.7 months, *p* < 0.001). Even within arm B, the overall survival of the patients underwent surgery was significantly longer than those who could not undergo surgery (31.3 vs. 15.9 months, *p* < 0.001). The results of the study indicated that long-term survival benefit could be obtained for patients with advanced gastric cancer through full-course comprehensive treatment and the tumor curative resection. In our cohort, the CRT with gastrectomy group had a significantly longer median overall survival than CRT only group (22 vs. 9 months).

In terms of the value of radiotherapy, it is usually used patients with stage IB to IIIB GC to downstage or downsize the primary site, increasing the possibility for radical resection (30). However, patients with stage IV GC has remote organ involvement, which is not appropriate for radiotherapy. Therefore, the main purpose was to control bleeding and improve quality of life (QoL) (31). However, concurrent chemoradiotherapy shows superiority to chemotherapy or radiotherapy alone in prolonging the survival of patients with metastatic GC (32, 33).

We acknowledge that our study still has some limitations. First, as a retrospective cohort study, although PSM was used to minimize the effect of the differences between the groups, selection bias is still a potential limitation of this study. Patients who underwent surgery were likely to have a potentially resectable disease when it was diagnosed, which might be one source of selection bias as well. In addition, due to the limitations of the SEER database, some information was not available to access, such as: removal of the metastatic sites, surgical margin status, D1/D2 node dissection, the chemotherapy regimen and the dose/field/intent of radiotherapy. In this study, the CRT with gastrectomy group had total or partial gastrectomy, which means the primary site was removed. However, it is unknown whether the metastatic sites were removed, which is a prognostic factor in stage IV GC as well. In terms of lymph node dissection extent, among the 146 patients who underwent surgery, the mean number of regional lymph nodes examined was 18.40, which was more than 15 lymph nodes minimum, as recommended by NCCN gastric cancer guidelines, to avoid stage migration (5). Therefore, considering the importance of these factors, the prolonged survival in the CRT with gastrectomy group that was observed in the current results should be interpreted with caution.

## Conclusions

In summary, stage IV gastric cancer remains a fatal disease. This study was a population-based study that revealed that, compared with CRT alone, CRT with gastrectomy may achieve a survival benefit in patients with metastatic GC. This indicated that selected metastatic gastric cancer patients may experience prolonged survival with primary tumor removal. Although its characteristics cannot be described currently, a further well-designed investigation is required to determine the best treatment strategy. Conversion therapy may provide a direction for the treatment of stage IV gastric cancer patients.

## Data Availability Statement

Publicly available datasets were analyzed in this study. This data can be found here: https://seer.cancer.gov/data/access.html.

## Author Contributions

All authors listed had made a great contribution to the work. LZ: came up with the concept/hypothesis, designed the study, and revised the manuscript. SL: collected and analyzed the data, drafted the manuscript. Finally, all the authors took responsible to the final manuscript and approved it for publication.

## Conflict of Interest

The authors declare that the research was conducted in the absence of any commercial or financial relationships that could be construed as a potential conflict of interest.

## Footnotes

^1^Surveillance, Epidemiology, and End Results (SEER) Program (www.seer.cancer.gov) SEERStat Database: Incidence - SEER 9 Regs Research Data, Nov 2017 Sub (1973–2015) < Katrina/Rita Population Adjustment> - Linked To County Attributes - Total U.S., 1969–2016 Counties, National Cancer Institute, DCCPS, Surveillance Research Program, released April 2018, based on the November 2017 submission.
